# A low-cost smart system for electrophoresis-based nucleic acids detection at the visible spectrum

**DOI:** 10.1371/journal.pone.0240536

**Published:** 2020-10-15

**Authors:** Eduardo Nogueira Cunha, Maria Fernanda Bezerra de Souza, Daniel Carlos Ferreira Lanza, João Paulo Matos Santos Lima

**Affiliations:** 1 Programa de Pós-graduação em Bioinformática (PPg-Bioinfo), Instituto Metrópole Digital (IMD), Universidade Federal do Rio Grande do Norte (UFRN), Natal, Brazil; 2 Departamento de Bioquímica, Laboratório de Sistemas Metabólicos e Bioinformática (LASIS), Centro de Biociências, UFRN, Natal, Brazil; 3 Instituto de Medicina Tropical do Rio Grande do Norte (IMT-RN), UFRN, Natal, Brazil; 4 Programa de Pós-graduação em Bioquímica, Centro de Biociências, UFRN, Natal, Brazil; 5 Departamento de Bioquímica, Laboratório de Biologia Molecular Aplicada (LAPLIC), Centro de Biociências, UFRN, Natal, Brazil; 6 Bioinformatics Multidisciplinary Environment (BioME), IMD, UFRN, Natal, Brazil; University of Glasgow, UNITED KINGDOM

## Abstract

Nucleic acid detection by electrophoresis is still a quick and accessible technique for many diagnosis methods, primarily at research laboratories or at the point of care units. Standard protocols detect DNA/RNA molecules through specific bound chemical dyes using a UV-transilluminator or UV-photo documentation system. However, the acquisition costs and availability of these devices, mainly the ones with photography and internet connection capabilities, can be prohibitive, especially in developing countries public health units. Also, ultraviolet radiation is a common additional risk factor to professionals that use electrophoresis-based nucleic acid detection. With that in mind, this work describes the development of a low-cost DNA/RNA detection smart system capable of obtaining qualitative and semi-quantitative data from gel analysis. The proposed device explores the visible light absorption range of commonly used DNA/RNA dyes using readily available parts, and simple manufacturing processes, such as light-emitting diodes (LEDs) and 3D impression. By applying IoT techniques, our system covers a wide range of color spectrum in order to detect bands from various commercially used dyes, using Bluetooth communication and a smartphone for hardware control, image capturing, and sharing. The project also enables process scalability and has low manufacturing and maintenance costs. The use of LEDs at the visible spectrum can achieve very reproducible images, providing a high potential for rapid and point-of-care diagnostics as well as applications in several fields such as healthcare, agriculture, and aquaculture.

## Introduction

Nucleic acid detection by gel electrophoresis is a ubiquitous laboratory routine procedure used in several fields, like genetics and molecular biology, biochemistry, and forensic science [1], and an essential step in extraction, cloning, and PCR workflows [2]. In the last decades, its increasing application allied with more accurate molecular diagnosis techniques has provided useful information on the general condition of patients, as well as contributing to the diagnosis and prognosis of various diseases [3]. Despite its simplicity, equipment and analysis costs are still high, especially in low resource settings (LRS) or for point-of-care (POC) applications.

The most used chemical dye for gel electrophoresis-based DNA/RNA detection is Ethidium Bromide (EtBr) [4]. This dye binds to DNA and, in the presence of ultraviolet (UV) light between wavelength 260 and 360 nm, fluoresces in orange-red range, in the length of 590 nm, being able to detect as little as 10 ng of DNA [5]. However, the use of EtBr presents several disadvantages, among them, is the fact that it is highly mutagenic and carcinogenic, as well as its requirement for UV light exposure for detection. Non-toxic fluorescent dyes are good alternatives to EtBr [6] nucleic acid staining, such as Methylene Blue, which has no mutagenic potential and no UV light need, although it is significantly less sensitive [7] and Sybr Green, which exhibits about the same sensitivity as the EtBr and can be visualized in the blue or UV light. More recently, there is also a line of more efficient and safe staining-ready gels, such as GelRed and GelGreen. However, they are expensive alternatives, and most laboratories, especially in developing countries, still use EtBr, despite its disadvantages.

The use of EtBr also popularized UV transilluminators for the visualization of nucleic acid gel electrophoresis. This device generates light at UV wavelengths to excite the fluorophore present in the gel, allowing the visualization of the molecule to which it is attached. However, these wavelengths are not the same for all DNA/RNA specific dyes. Therefore, most equipment available on the market is not specific for all applications in research and clinical laboratories. Although it is a conceptually simple instrument, transilluminators have a high cost, about U$ 500.00 to 2,000.00, even without adding photodocumentary capabilities, which is usually only justified by its application. Moreover, camera-coupled transilluminators usually need to be connected to a computer and positioned on a transilluminator or have an integrated chamber for the acquisition of images. The associated costs, coupled with its low portability, often make it infeasible to use in places with limited structures such as LRS laboratories or POC health units in developing countries.

With the advancement of technology and its availability, it is possible to develop systems that previously had a very high cost at very competitive prices in the market. Also, the growth of "do it yourself" mentality, the increasing accessibility to top-end technologies, and the Internet of Things (IoT), all have broken paradigms of connectivity and development of electronic products [8]. The most significant search today in all areas is for integrated, portable, low energy, automatic, and low-cost smart devices. In the laboratory environment, simple technologies combined with excellent engineering had allowed a reduction of up to 20 times in the values of some equipment. At present, easily accessible materials can allow the development of thermal cyclers for PCR [9], electrophoresis apparatus [10], and nucleic acid direct detection systems [11–14]. With that in mind, the aim of the present work was the development of a low-cost DNA/RNA detection smart system capable of obtaining qualitative and semi-quantitative data from gel electrophoresis analysis from different available dyes. With easily accessible material and technologies, as light-emitting diodes (LEDs), miniaturized computing, and 3D impression, and also using a smartphone and custom software, it was possible to obtain a capable device to substitute a conventional commercial UV-transilluminator in several applications.

## Materials and methods

**Fig 1:**
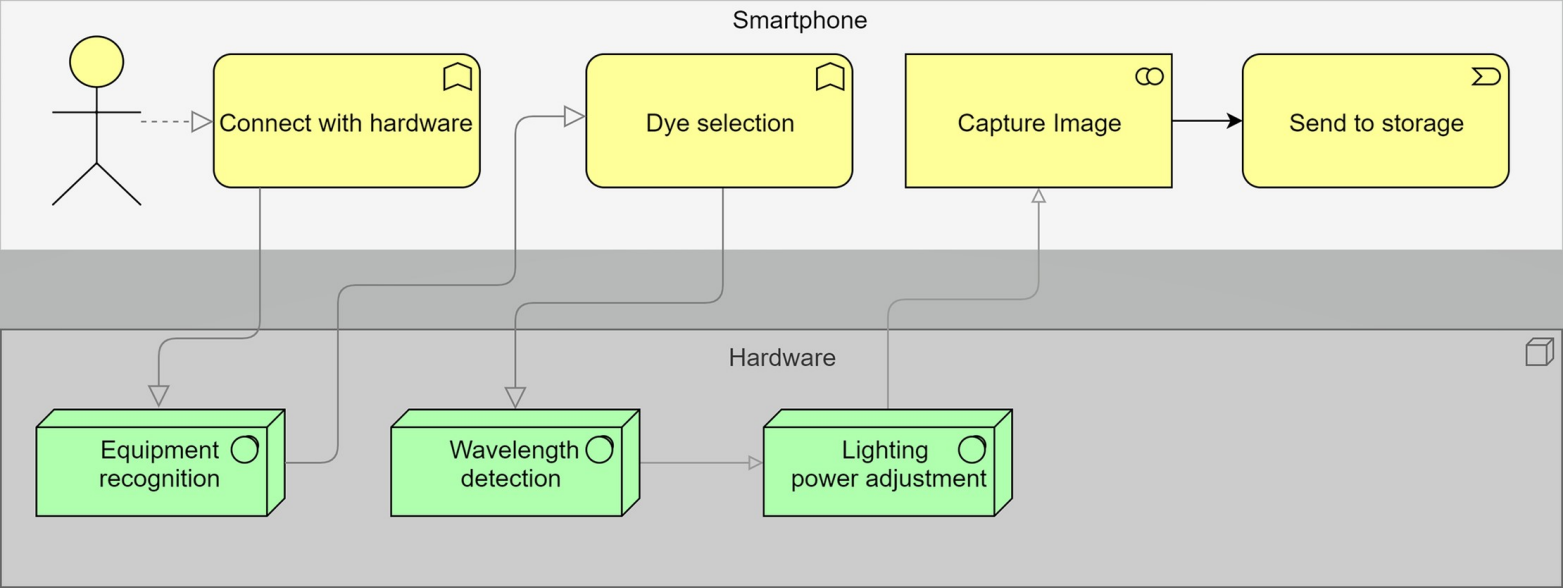
A schematic diagram demonstrating an overview of the model's project. The diagram also describes the tasks executed by the smartphone software (yellow boxes) and by the hardware (green boxes), and their respective workflow.

The project of the device comprises two parts, software development and the system hardware *per si* (Fig 1), connected by Bluetooth protocols.

### Hardware

The system hardware consists of a microcontroller platform with wifi and Bluetooth connections. It also includes an adaptable controller to various dye types, selecting the wavelength emitted by the lighting system for the type of dye used in the experiment, summarized in “Table 1”. Lighting intensity control is also implemented in hardware to optimize image capture, as shown in [15].

**Table 1:** Excitation and emission wavelengths for commonly available commercial dyes.

*Fluorochrome visible spectrum*	*Excitation (nm)*	*Emission (nm)*	*Dyes*
Blue	343–431	455–483	*DAPI*
			*Hoechst*
			*SYTOX*
			*Hoechst*
Green	504–509	509–533	*YOYO-1*
			*SYTOX green*
			*TOTO 1*
			*Sybr-Green*
Yellow	445–547	570–575	*SYTOX orange*
			*Chromomycin*
			*Mithramycin*
Red	493–536	617–620	*Propidium iodide*
			*Ethidium bromide*
			*GelRed*

Reference guide to the detection of several dyes using LEDs under the visible light spectrum.

#### Lighting system

For the excitation of the dyes, the system uses fourteen 3 W RGB LED chips (3x1W, with six K1763 terminals), with a coupled heat sink, divided into three separate sets. Each set has unevenly powered red, blue, and green LEDs. This division in the electric power does not happen in the same way (as will be described in the elaboration of the control circuit of the led) since the voltage of the red LED matrix is smaller. This uneven power division compensated the amount of lux emitted by the red LED and permitted an equal luminous power. The wavelength range of these LEDs is 620–630 nm, 520–530 nm, and 460–470 nm, for red, green, and blue, respectively. S1 File describes the LEDs full specification.

#### Electronic circuit

For the design of the LED's control circuit, we used a pulse width modulation (PWM) system. To obtain control and optimize the lighting system according to the arrangement of the box, we used one PWM for each set of LEDs. The LED chip RGB consists of three parallel LEDs, mounted with a common anode, which need the same voltage (24 V) and current (600 mA) for each color matrix in each LED. The drive circuit has taken into account the switching speed for pulse modulation so that it is possible to obtain the full-color spectrum within the visible, and there is no excess energy dissipation by Joule effect [16], not compromising the durability of the card and components.

**Fig 2:**
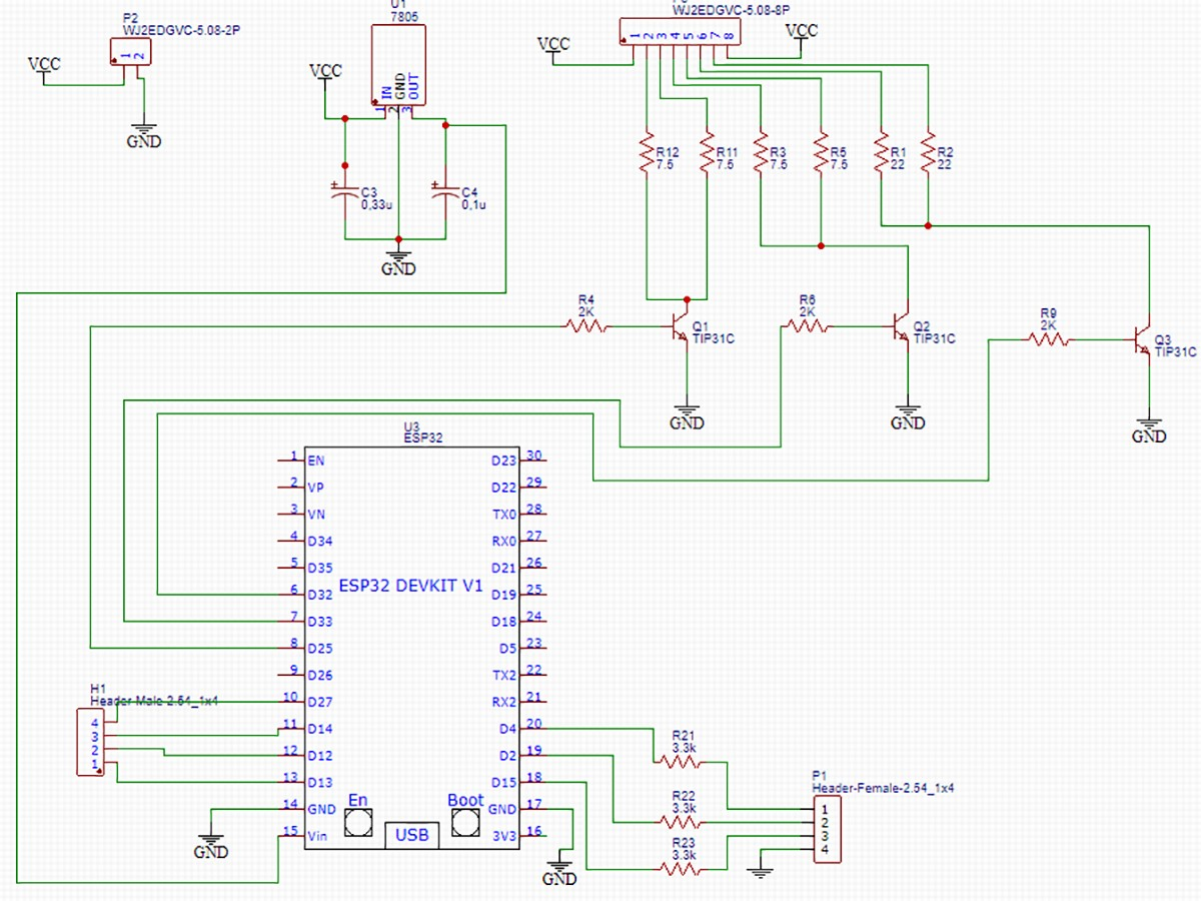
Diagram representing the system's electric circuit, describing the component dimensioning and best arrangement, as well as the respective microcontroller inputs and outputs.

The microcontroller used was the ESP32 (Espressif) [17], operating at 0.8 mA current at the port, well below its maximum capacity of 12 mA. The system's project also included touch screen buttons and signal operation LEDs mounted to an auxiliary circuitry. For controlling the LED cooling system, aluminum heat sinks were mounted directly on the LED board. The circuit contained correctly dimensioned components to avoid overload of the LED chips during use. (Fig 2) describes the circuit project and shows the components already sized.

#### 3D printing

**Fig 3:**
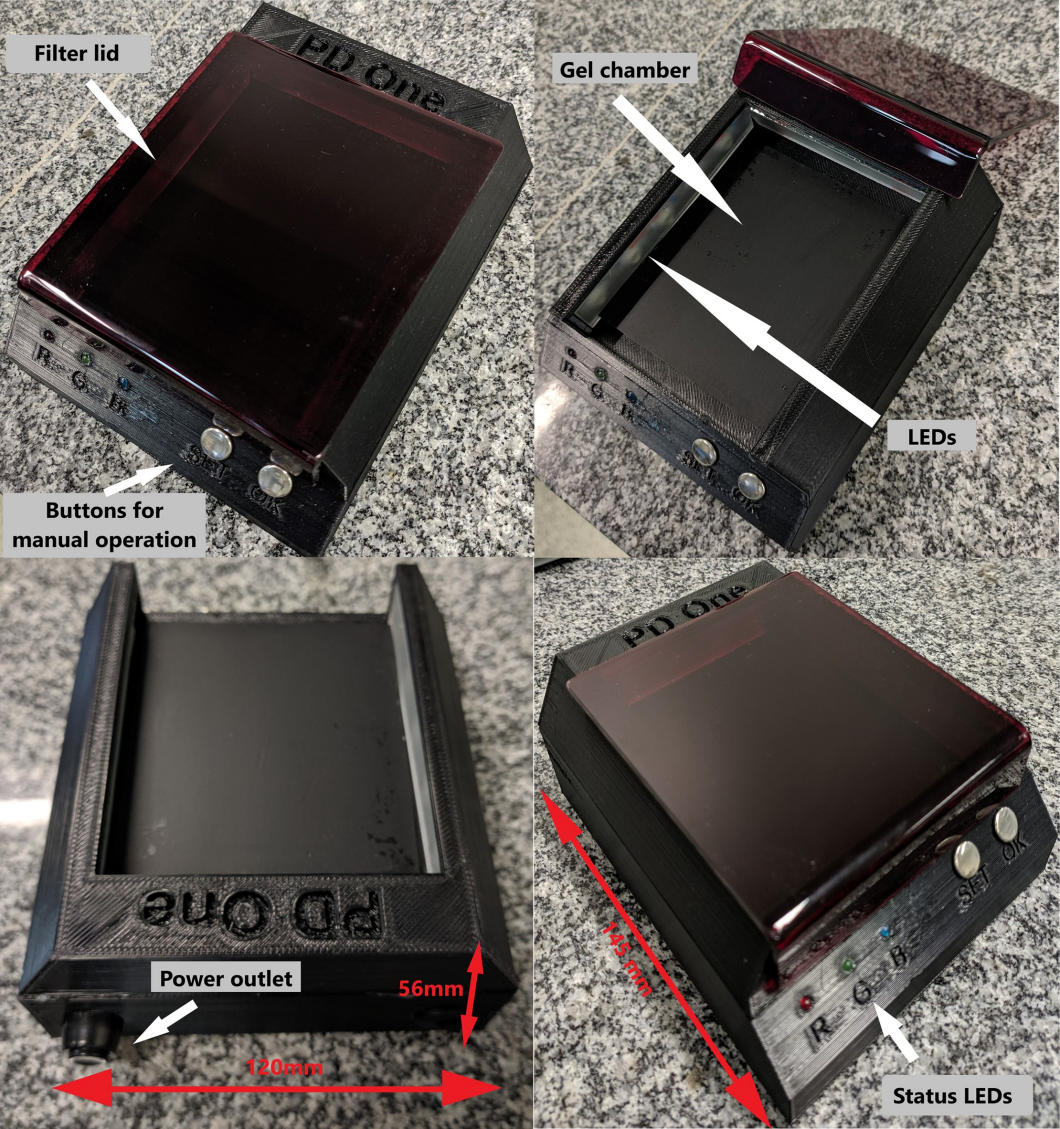
Final assembly of the system after 3D printing. White arrows indicate the filter lid, the status LEDs, the gel chamber, the power outlet, and the "SET" and "OK" buttons for manual operation of the system.

To maintain a low cost and achieve a straightforward reproduction, we opted to use 3D printing for the development of the platform's on-site photographic documentation system. (Fig 3) shows the final prototype. The calculation of the size and angles for the smartphone support depends on its camera's specifications and must guarantee that the entire illuminated area is covered. In the tested prototype, we used the following measures:

○ The measures of the support.
  ■ Height 56 mm, width 120 mm, and depth 145 mm.
  ■ Minimum distance for image capture: 90 mm.
○ Xiaomi MI 9 SE smartphone, the sensor Sony IMX586 Exmor RS, there is three cameras 48 MP + 16 MP + 12 MP, resolution 8k x 6k pixel, sensor size 1/2 " + 1/3 " + 1/3.6 " and aperture size F 1.75 + F 2.2 + F 2.2. For this experiment, we used only the 48 MP camera, but a minimum setting of 12 MP is sufficient for viewing the bands on the proposed device.

S1 File includes simple calculations for the support's measures, for standard cameras available. The constructed prototype can accommodate gels in any range according to the calculations presented. The inclusion of the SET and OK buttons allows the system's operation as a simple transilluminator, without using a smartphone, though power and wavelength selections are software-managed only. S1 File describes the CAD drawing model for 3D printing.

### Software

The system includes two operating software. The ESP32 microcontroller software, written in C programming language, has the function of controlling the pulse width modulation system of the LED chips, the communication with the smartphone via Bluetooth low energy (BLE), and the touch buttons. The Android smartphone application is responsible for connecting to the ESP32 microcontroller via Bluetooth, as well as managing the type of dye used, its frequency (color) and intensity with the emitted light, and image capture. There is also an included function to execute an automated wavelength scanning process. At this step, the user has an option to automatically or manually validate the captured image. Another function implemented in smartphone software along with hardware is image capture optimization [18, 19], to synchronize the camera frequency with the pulse cycle of the used LEDs. After the image validation process, the software sends data for storage or remote analysis. This application, developed in an online block programming environment at Thunkable®, also controls the included photography and sharing functions.

### Sample preparations and gel electrophoresis

The DNA used was the PCR product of a conserved fragment of 18S DNA from the shrimp (*Penaeus vannamei*) genome. This fragment has a similar pattern in almost all decapod with an 848 bp amplicon, corresponding to the sequence region 352–1200 of the 18S rRNA. The used primers were: 143F 5'-TGC-CTT-ATC-AGCTNT-CGA-TTG-TAG-3' and 145R 5'-TTC-AGN-TTT-GCA-ACC-ATA-CTT-CCC-3' (N represents G, A, T or C) [20]. PCR's reactions had a final volume of 75 μL containing: 0.25 μM from each primer, 2.5 mM MgCl_2_, 2.5 mM of each dNTP, 1 U GoTaq® DNA Polymerase (Promega), and 6 μL of the template DNA. PCR products were quantified using the QuantiFluor One dsDNA system. The concentration of the PCR final product was 38 ng/μL. Eight samples from seven serial dilutions of the PCR final product (38.00, 19.00, 9.50, 4.75, 2.38, 1.19, 0.59, 0.30 ng/uL) were prepared and later observed in three distinct 1% agarose gels, using SYBR Green®, GelRed®, and ethidium bromide as chemical dyes. 6 μL of each diluted sample were applied to the gel’s wells, totalizing the following DNA quantities: 228, 114, 57, 28.5, 14.28, 7.14, 3.54, and 1.8 ng.

### System calibration

**Fig 4:**
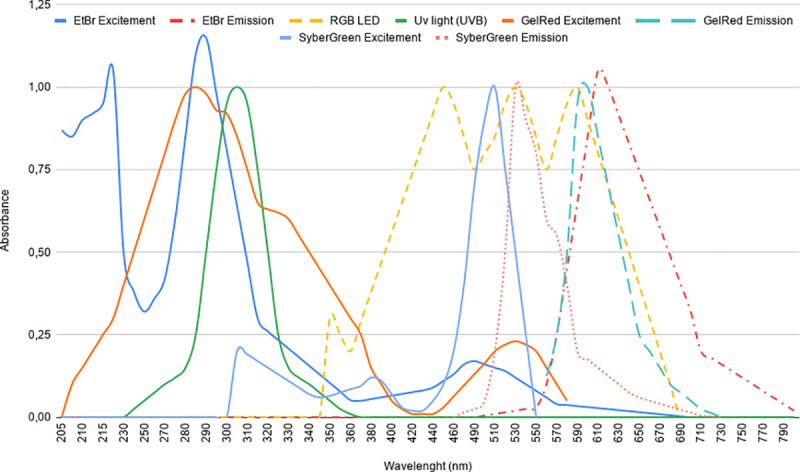
Plot representing the emission and excitation of the used dyes. Wavelengths are in the range of 205–790 nm (UV until visible). Full and dashed lines represent the emission and excitement wavelengths, respectively.

In order to obtain the best viewing point for each of the dyes, we performed a calibration process using a tracking module implemented in the device's software, which scans the entire region of the visible spectrum with an automatic increment of 10 nm (Fig 4). After that, we compared the results with the spectroscopy provided in each dye manual to ensure the use of its highest excitement regions. For the calibration process, ten independently prepared gels were used for each type of dye, with a more thorough scan for the dye with higher excitation within the UV region. “Table 2” describes the power levels for each color LED and the RGB values used for each dye.

**Table 2:** LED Power levels (%) for each color and the RGB values used for EtBr, gelRed, and Sybr Green dyes.

Dye	LED RGB						
	nm	LED Red		LED Green		LED Blue	
		Pot.	RGB	Pot.	RGB	Pot.	RGB
ETBR	490	0%	0	60%	153	60%	153
gelRed	530	10%	26	65%	166	40%	102
Sybr Green	500	20%	50	60%	153	30%	77

Power levels are expressed in percentage of the total power of each color in the LED chip.

### Image analysis

During visualization within the system, all the gels were photographed with the coupled Xiaomi MI 9 SE smartphone (Android Version 10). For comparison reasons, the gels were also visualized and photographed using a 302 nm wavelength standard transilluminator. The resulting raw images (without software treatment) were cropped, then analyzed and compared in ImageJ software [21]. From ImageJ densitometric analyses we calculated the Lower Limit of Detection (LLD) values, as described in [22].

## Results and discussion

### Assembly costs

We aimed to develop a complete device for DNA/RNA analysis using the electrophoresis technique, commonly used today in many research and health diagnostic laboratories. “Table 3” describes the cost of each part. The average assembly cost was US$ 53.76, without including smartphone cost, which is pronouncedly cheaper than every commercially available system with similar functionalities. Rather than substituting a camera-coupled transilluminator entirely or for all its applications, this system is supposed to aid rapid and early electrophoresis-based diagnostics methods, especially in developing countries, either in POC units or at field or LRS conditions. Since there are different sizes of electrophoresis gels and their respective applications, the project presented here is easily modifiable to accommodate larger gels and can even be adapted to the construction of devices that use capillaries system for detection. Besides, by using a smartphone internet connection, results can be remotely sent to further analysis at reference health centers or laboratories. Regarding its low costs and uncomplicated manufacturer process, another potential application of this system is for science education at public schools, which usually have a low budget for scientific equipment [23].

**Table 3:** Parts and materials used in the manufacture and assembly of the system, and respective costs (not including the smartphone price).

*Part/Material*	*Quantity*	Cost (US$)*
ESP32 microcontroller (Expressif)	1 plate	3.76
LED RGB 3W (K1763)	14 LED chips	8.00
Acrylic (3mm thick red acrylic)	80 cm^2^	2.00
3D printer PLA filament (black color, 1,75 +-0,05 mm)	300 g	9.00
Various Electronics components (resistor, transistors, capacitors, printed circuit board and connectors)	-	10.00
Various Miscellaneous Parts (masking tape and separators)	-	1.00
3D printing (print time 13 h)	13 h	20
**Total cost (without smartphone)**		53.76

*Average costs of the required materials. These costs refer to a system able to accommodate gels sizes from 80 cm^2^. The material acquisition occurred between the dates 08/01/2019 and 11/30/2019.

### Software management and dye detection

**Fig 5:**
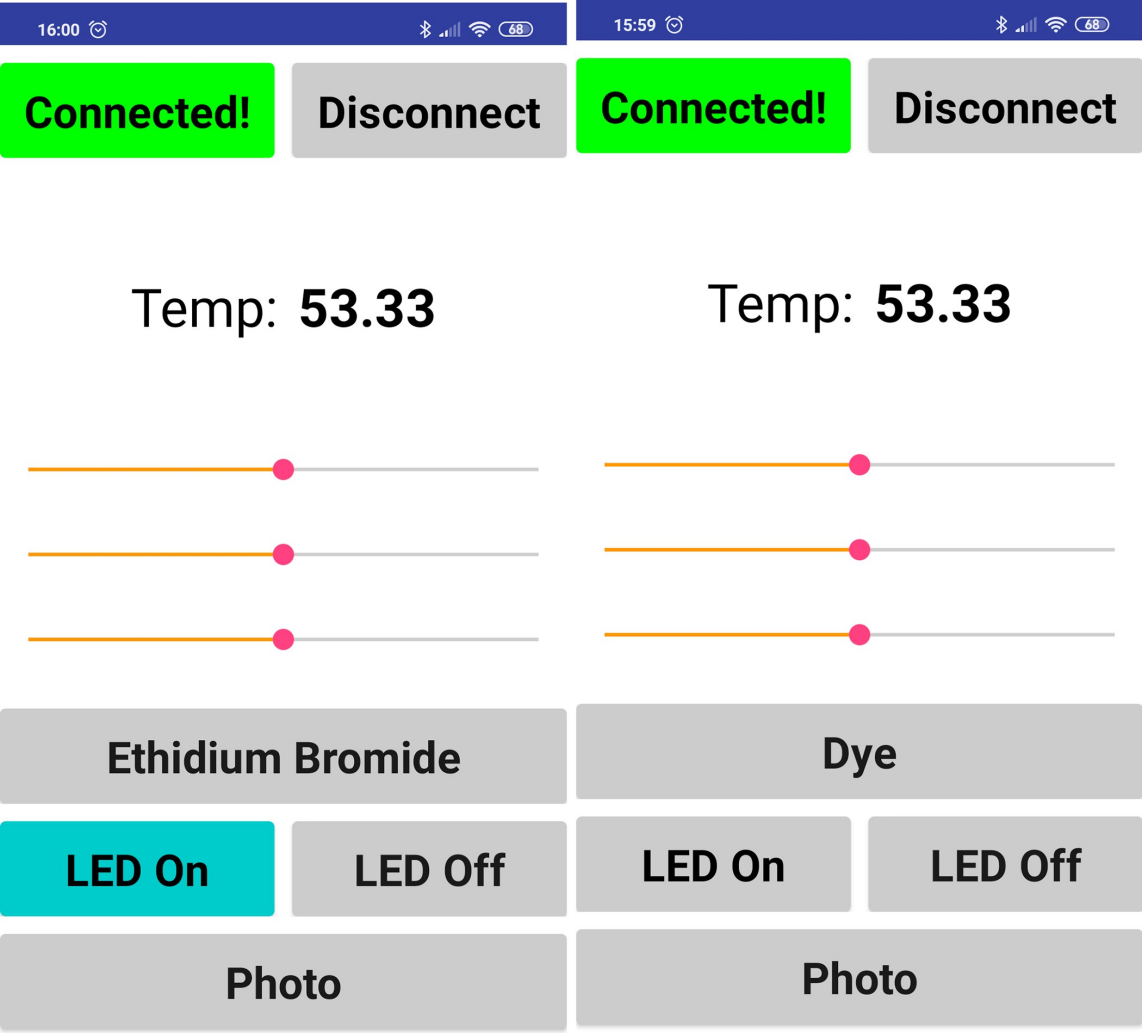
Screenshots of the smartphone software. In the software, it is possible to monitor the device temperature and to make the fine-tune adjustments for dye selection. Initial screen (right). Preset for using ethidium bromide as a dye (left).

Using RGB LEDs, we were able to get all colors within the visible spectrum by combining the primary colors, red, green, and blue. Therefore, we got a piece of equipment that can excite any commercially available dye, since it has a known visible-light emission wavelength range. As can be evidenced in (Fig 4), most DNA/RNA specific dyes have a low but sufficient excitement within the visible spectrum. For EtBr, a commonly used dye in molecular analysis laboratories, due to its low cost, we achieved excitation, emission, and detection without the use of UV light. Although EtBr has a higher excitement within the UVB (280–315 nm) region, conventional UV lamps used in transilluminators have a considerable displacement, not taking full advantage of this peak region [24], which is still significantly higher than the excitement region within the visible spectrum [25]. However, our system can excite EtBr molecules at its peak visible absorption, around 480 nm, by using high-luminance LEDs, which obtain a better excitement optimization at this range and partially compensate for this energy difference. The developed smartphone associated software has a screen for selecting the dye used for detection (Fig 5). Afterward, it automatically sets LED colors to maximize dye-specific excitement. Also, since the tracking module scans the entire visible region, the adjustment of the system is possible to ensure the use of dyes' highest excitement regions. Therefore, calibration is essential for further optimization and also for the correct registration of wavelengths for each dye in the smartphone software.

### Sensitivity analysis

To evaluate the visualization potential of the developed system, we used the same amplified DNA sample dyed with three distinct and commonly used dyes (EtBr, GelRed, and SYBR Green), comparing the results with the visualization using a standard UV-transilluminator. By using smartphone software communication (Fig 5), we selected the highest point of excitation from each dye within the spectrum for the RGB LED, as shown in (Fig 4). Therefore, the used excitation wavelengths were 490 nm (Cian), 500 nm (Green), and 530 nm (Green), for EtBr, GelRed, and SYBR Green, respectively.

**Fig 6:**
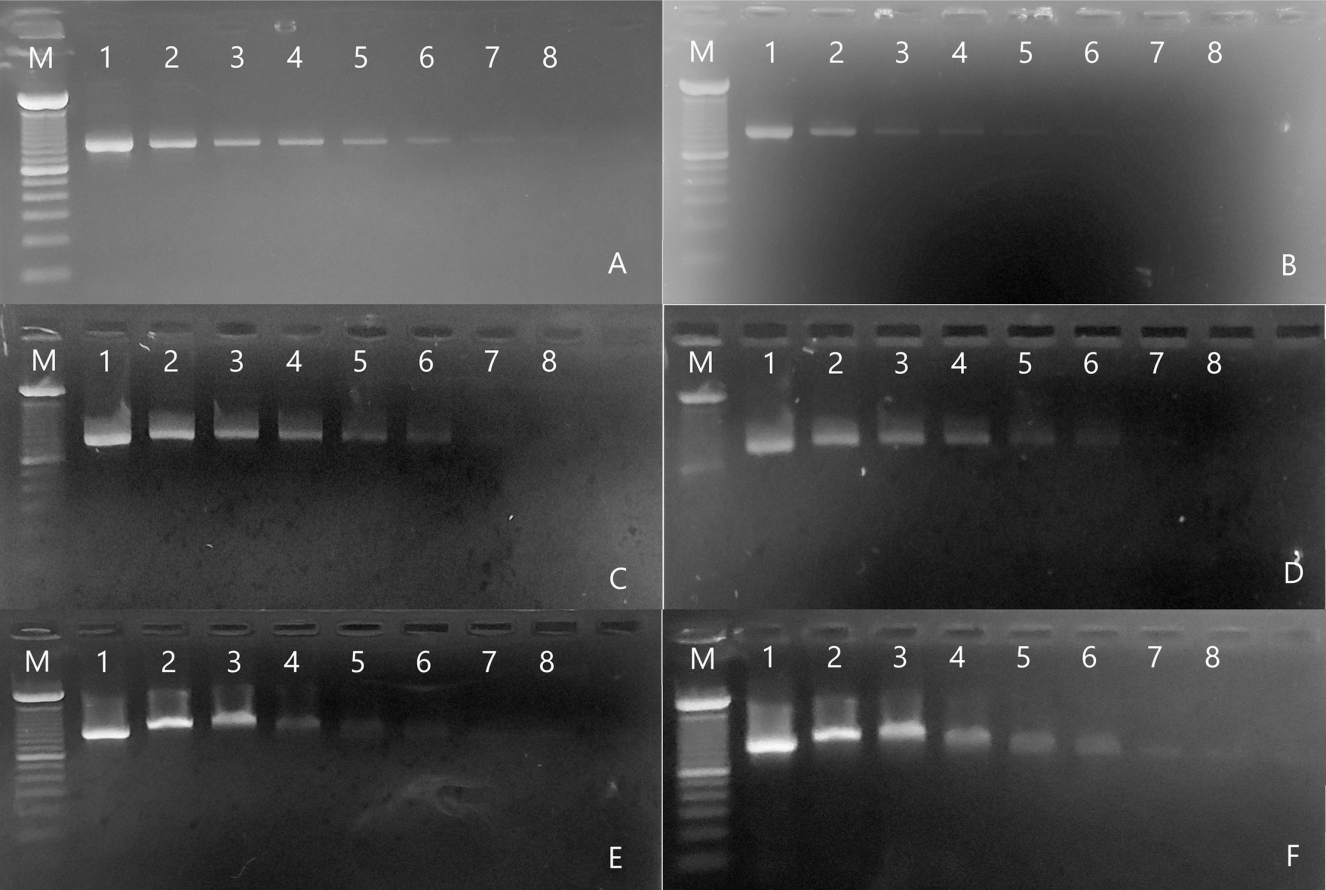
Agarose gel electrophoresis images from three different dyes. Ethidium bromide (A, B); GelRed (C, D); and SYBR Green (E, F). Photos obtained from the use of a conventional UV-transilluminator are on the left panel (A, C, and E). Images captured from the proposed device are on the right panel (B, D, and F). All photos underwent the same image treatment in the ImageJ software [21] so that there was no advantage for comparative analysis. Samples in the gel lanes are serial dilutions of the PCR final product (see Material and Methods). M–HighRanger 1 Kb DNA Ladder molecular marker (Norgen Biotek Corporation); DNA quantities: 1–228 ng; 2–114 ng; 3–57 ng; 4–28.5 ng; 5–14.28 ng; 6–7.14 ng; 7–3.54 ng; 8–1.8 ng).

**Fig 7:**
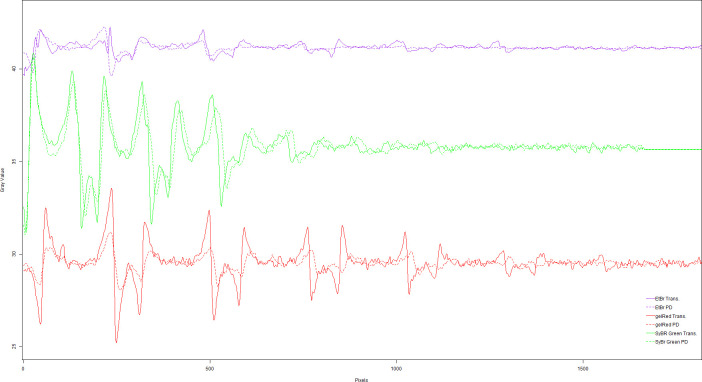
Gel images analysis. Analysis of intensity quantification (by pixel density) of the agaroses gel images obtained with a conventional transilluminator (full lines—T) and our proposed system (dashed lines—PD), with the three used dyes: Ethidium bromide (purple lines); GelRed (red lines); SYBR green (green lines).

As can be seen in Fig 6A and 6B, the visualization of EtBr stained bands is more definite and precise in the photos taken on the UV-transilluminator. As discussed before, this is due to the high adsorption potential of this dye within the UV region (Fig 7). By analyzing the plot of the grayscale difference of bands' densitometry (Fig 7) from the proposed device, we perceived that it is not possible to view the samples at low concentrations. The lower limit of detection (LLD) values for the EtBr dye were 29.34 and 45.06 ng, using the UV-transilluminator and our LED system, respectively. Since reported EtBr sensitivity values in agarose gels range from 1.0–10.0 ng of DNA [26–28], the LLD of the proposed system is adequate for several EtBr applications.

Visually, we observed similar results for the GelRed staining Figs 6C, 6D and 7. Although this visual difference, the estimated LLD for GelRed from our system (47.58 ng) was lower than the calculated value from the UV-transilluminator detection (65.22 ng). This was probably due to the fact that GelRed excitation and detection are more uniform and proportional under visible light (Fig 7; S1 File). However, both detection systems presented higher LLD values than the reported sensitivity values for GelRed dye [27]. Despite the difficulty of visual evaluation of the more diluted EtBr and GelRed-dyed bands in photos taken by using the proposed device, it does not invalidate its use for these dyes. Low concentrations are usually not recommended for qualitative or semi-quantitative analysis [29]. Additionally, optimization of protocols and sample concentrations can compensate for this potential weakness, with the advantage of using a device that is safer than conventional UV-transilluminators, since it does not emit UV radiation.

Nevertheless, the device surpasses conventional UV-transilluminators for the use of SYBR green-based protocols. SYBR-stained DNA bands were more brilliant and pronounced in photos taken under the illumination of visible LEDs Figs 6E, 6F and 7. The system takes advantage of the high absorption potential of this dye within the visible region, as noticed by its significantly lower LLD values than the ones from the UV-transilluminator (42.36 ng x 101.58 ng, respectively). Under this chemical staining, it is indeed difficult to evidence more diluted samples under the transilluminator radiation. Although it also has its toxic effects [30], SYBR-green and GelRed have surged as a safer alternative for EtBr nucleic acid staining [27, 31, 32], with the plus of having a more uncomplicated decontamination process [33].

The use of excitation and emission filters could reduce the background noise and enhance the proposed device's detection capabilities, as evidenced by [11]. However, specific filters addition would increase the device's costs and the overall project complexity. We overcame this by using a red acrylic lid as a filter since all dyes have an emission above 500 nm. Without filter addition, our system can detect other chemical dyes, which remains an accessible, low-cost option for LRS in emerging countries, especially for qualitative molecular tests.

### Functionalities and features of the system

“Table 4” summarizes the main features and specifications of the proposed system compared to conventional commercial transilluminators. Our system is a low-cost example of a new generation of transilluminators that aims to apply the concepts of IoT in laboratory equipment. The insertion of the technology has the clear objective of increasing security both in not using UV radiation but also avoiding the user's contact with the transilluminator, by the use of a smartphone as a control for dye adjustment and image capture and processing.

**Table 4:** Features and specifications comparison between a conventional commercial transilluminator and the proposed device.

*Part/Material*	*Commercial Transilluminator*	*New Transilluminator*
UV radiation	x	
Adjust wavelength according to dye		x
Wireless operation		x
Estimated lamp life	1,000 h	50,000 h
Energy consumption	1.5 kW	0.15 kW
Weight	7 kg	0.4 kg
Cost	US$ 500–2000	US$ 53.76

## Conclusion

The low-cost smart detection system here presented can substitute a conventional UV-transilluminator in several electrophoresis-based applications, allowing the detection of nucleic acid bands staining with three commercial dyes. Besides the low-cost, other advantages of our system are the maximization of dye-specific excitement under the visible light spectrum, portability, and connectivity. The performed analysis also demonstrated that, in some cases, the device could achieve better results than a standard UV-transilluminator for the detection of SYBR green-dyed DNA bands. These features, allied to its easily accomplishable construction, confirm the potential use of this device in low resource settings (LRS) or for point-of-care (POC) and educational applications.

## Supporting information

S1 File Supplementary information. Document file describing the calculations for the support's measures for standard cameras available, the LED specifications, the CAD drawings for the system and linear regression plots from the densitometric analyses.
(PDF)

S1 Raw images (PDF)
