# Supplementary material for: A low-cost smart system for electrophoresis-based nucleic acids detection at the visible spectrum
Source: PLoS One. 2020 Oct 15;15(10):e0240536. doi: 10.1371/journal.pone.0240536 (PMC7561130; doi:10.1371/journal.pone.0240536)
Supplement: S1 File — Document file describing the calculations for the support's measures for standard cameras available, the LED specifications, the CAD drawings for the system and linear regression plots from the densitometric analyses. (PDF) [file pone.0240536.s001.pdf]

Supporting Information

Data analysis - Densitometry analysis and calibration curves

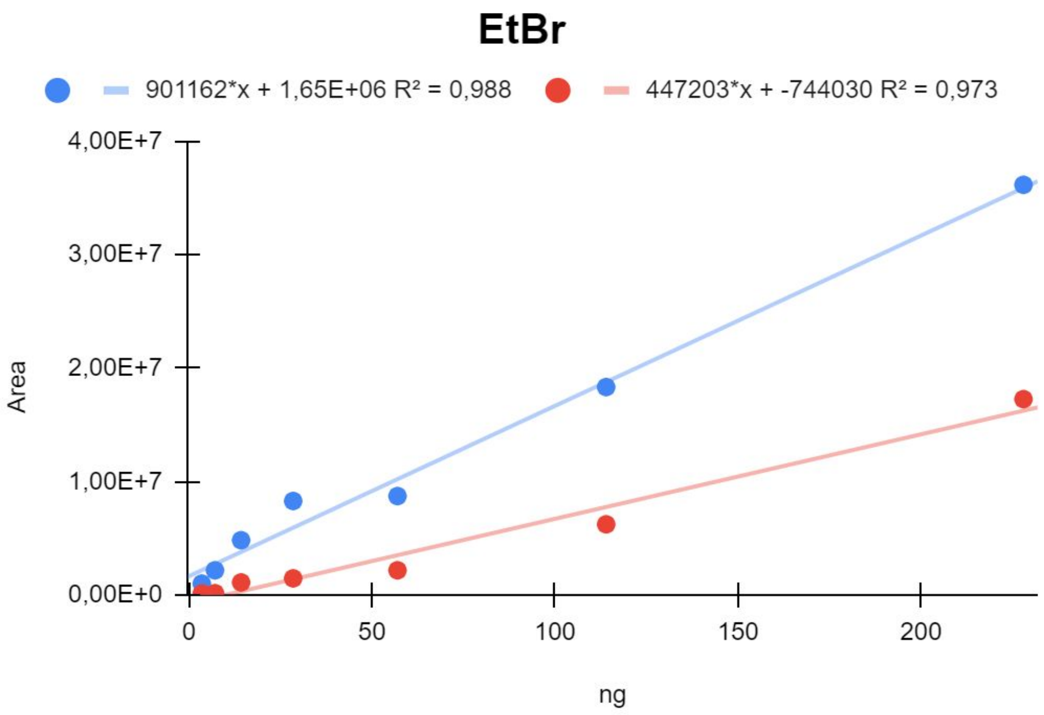

Fig S01. Linear regression plots of the densitometry analysis for the EtBr dye, for the UV-transilluminator (blue line and dots), and the proposed device (red line and dots). Ethidium Bromide's LLD values for the UV-transilluminator and the proposed system are 29.34 and 45.06 ng, respectively.

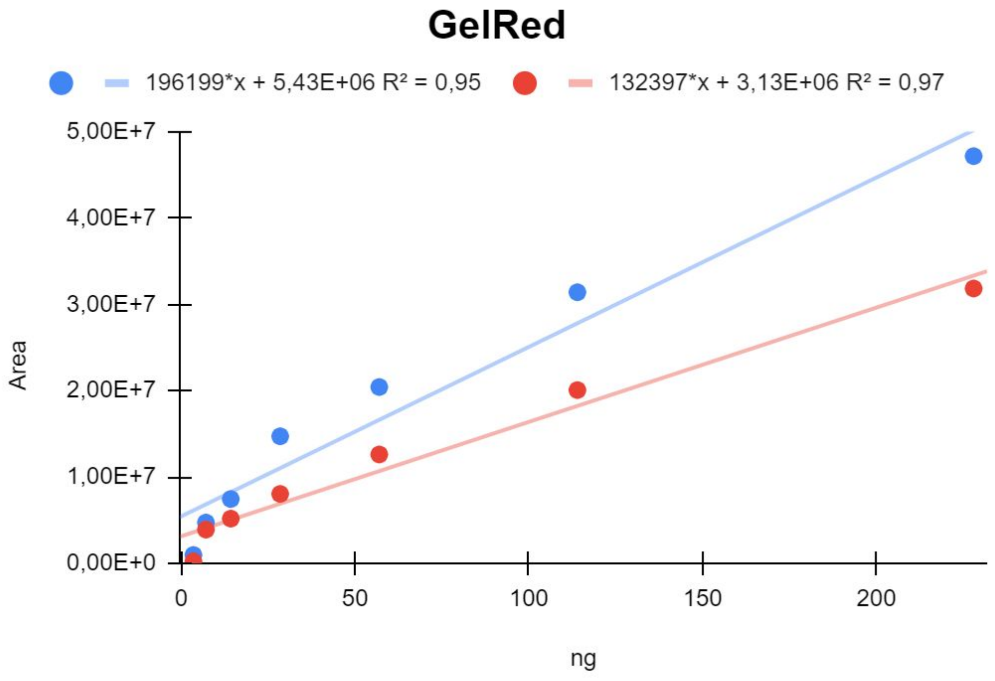

Fig. S02. Linear regression plots of the densitometry analysis for the gelRed dye, for the UV-transilluminator (blue line and dots), and the proposed device (red line and dots). gelRed's LLD values for the UV-transilluminator and the proposed system are 65.22 and 47.58 ng, respectively.

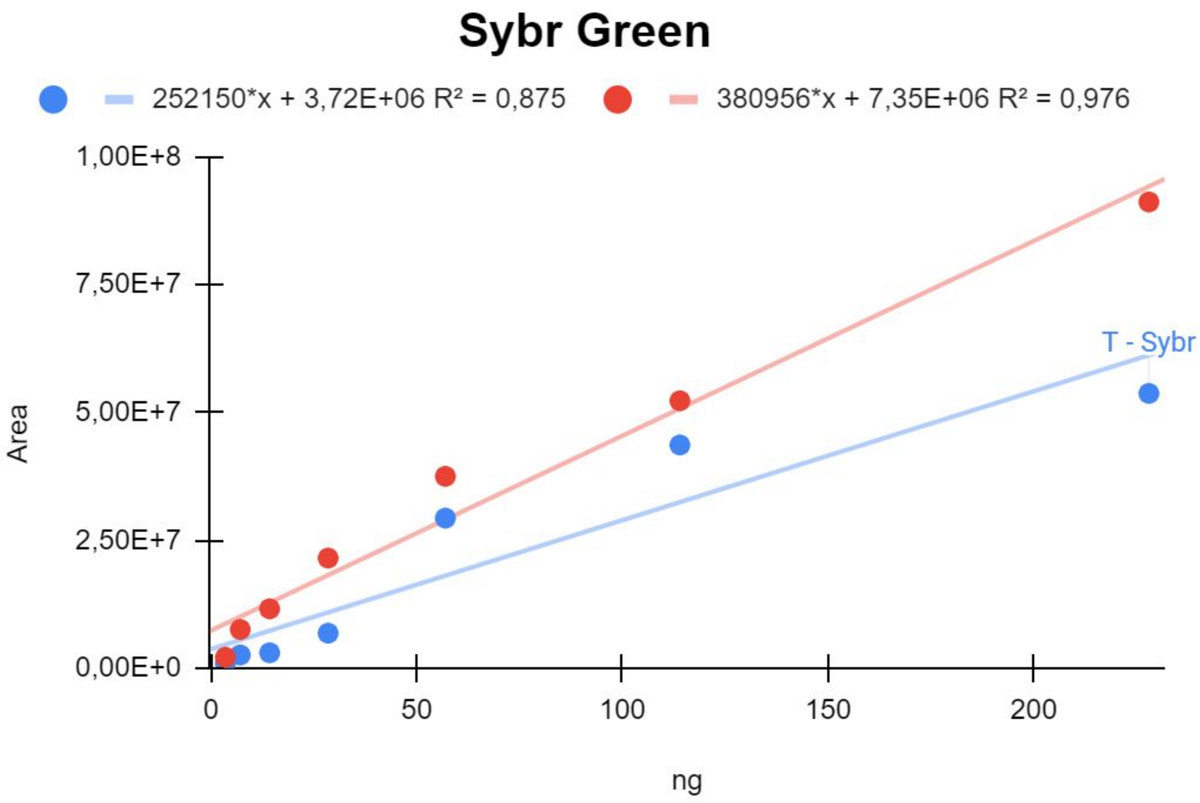

Fig. S03. Linear regression plots of the densitometry analysis for the Sybr-Green dye, for the UV-transilluminator (blue line and dots) and the proposed device (red line and dots). Sybr-Green's LLD values for the UV-transilluminator and the proposed system are 42.36 and 101.58 ng, respectively.

Calculations for equipment sizing

For the prototype design, a table was made based on tests of absorption and reflection of light at the illuminated area. All dimensions were predicted according to the area to be occupied by the gel. In Fig 04, 05, and 06, we can see the prototype sized for a 70 x 80 mm gel. All measurements used in the formulas are in millimeters (mm).

- $Number\ of\ Leds\ =\ abs\left[\frac{With + Deph\ x\ 2}{20}\right]$
- $Led\ light\ angle\ of\ incidence\ = tg\left(\frac{gel\ width}{4}\right)^{-1}$
- $Minimum\ distance\ for\ image\ capture\ = \frac{tg\frac{\theta}{2}}{area}$
- $Equipment\ depth\ = gels\ depth\ + 45$
- $Equipment\ width\ = gels\ width\ + 40$
- $Equipment\ height\ = 56$

where:

$\theta = camera\ angles$;

$Area = gel\ area$.

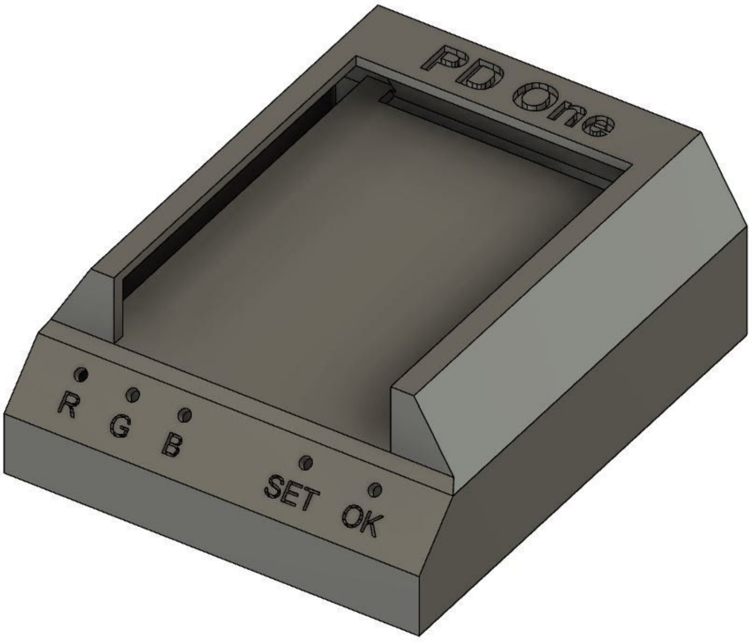

Fig S04. Complete box model, after assembly.

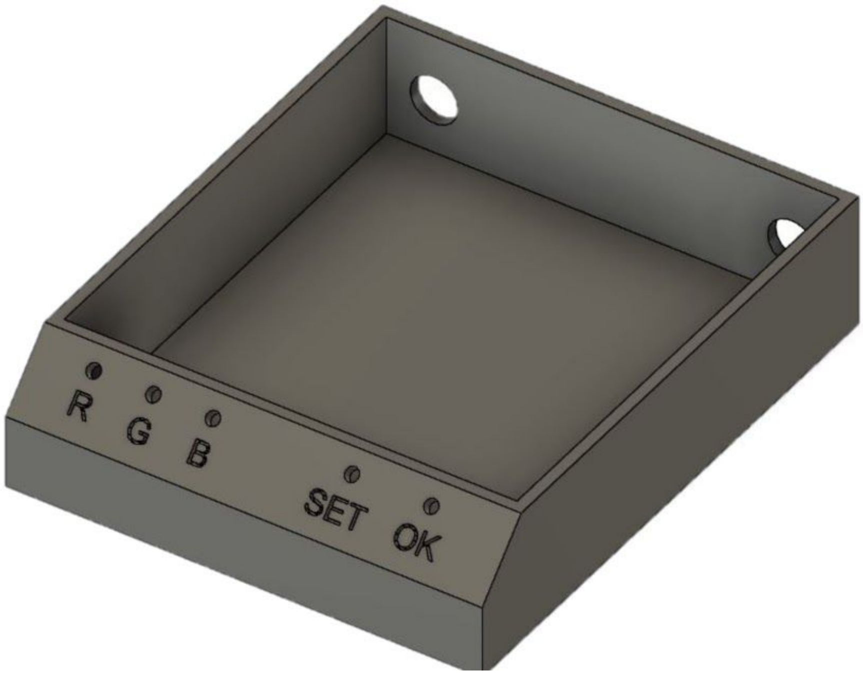

Fig S05. Bottom of the box with space for circuit allocation.

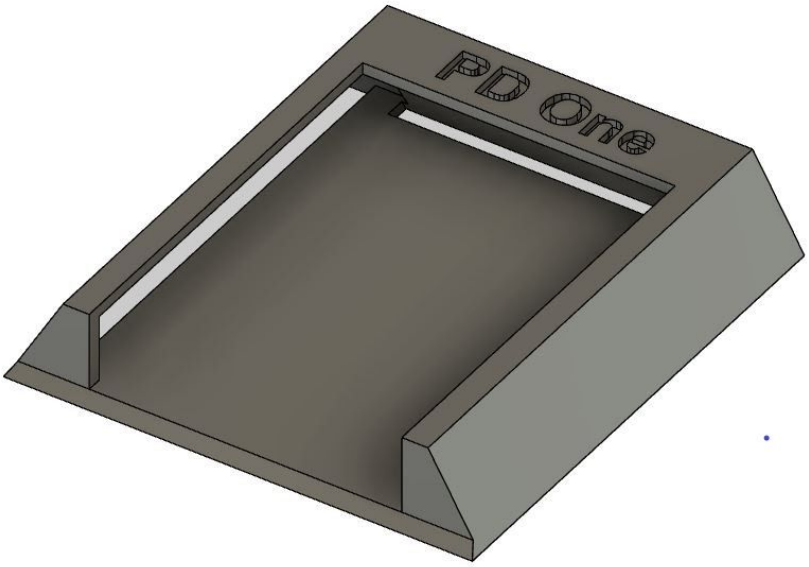

Fig. S06. Top of the box with side space with the inclination for the allocation of LEDs.

Circuit Design

The circuit design has the aim to obtain the lowest possible current at the source. For this, we make a series of parallel arrangements with the LEDs (Fig 07), respecting the maximum current allowed by each LED. Another purpose of the circuit is to compensate for the difference between the luminous power versus the electrical power of the LEDs to obtain the best balance for capturing images.

The LED used in the experiment was the LED 3w RGB (REF: K1763) and has the following specifications:

- Power: 3W (3 x 1W)
- Operating voltage: (R: 2.0 - 2.4V, G: 3.2 - 3.4V, B: 3.2 - 3.4V)
- Operating current :(R: 350 mA G: 350 mA B: 350 mA)
- Luminous Flux: (R: 40 - 50 Lm, G: 80 - 95 Lm and B: 80 - 90 Lm)
- Wavelength: (R: 620 - 630 nm, G: 520 - 530 nm, B: 460 - 470 nm)
- Duration: 50,000 hours

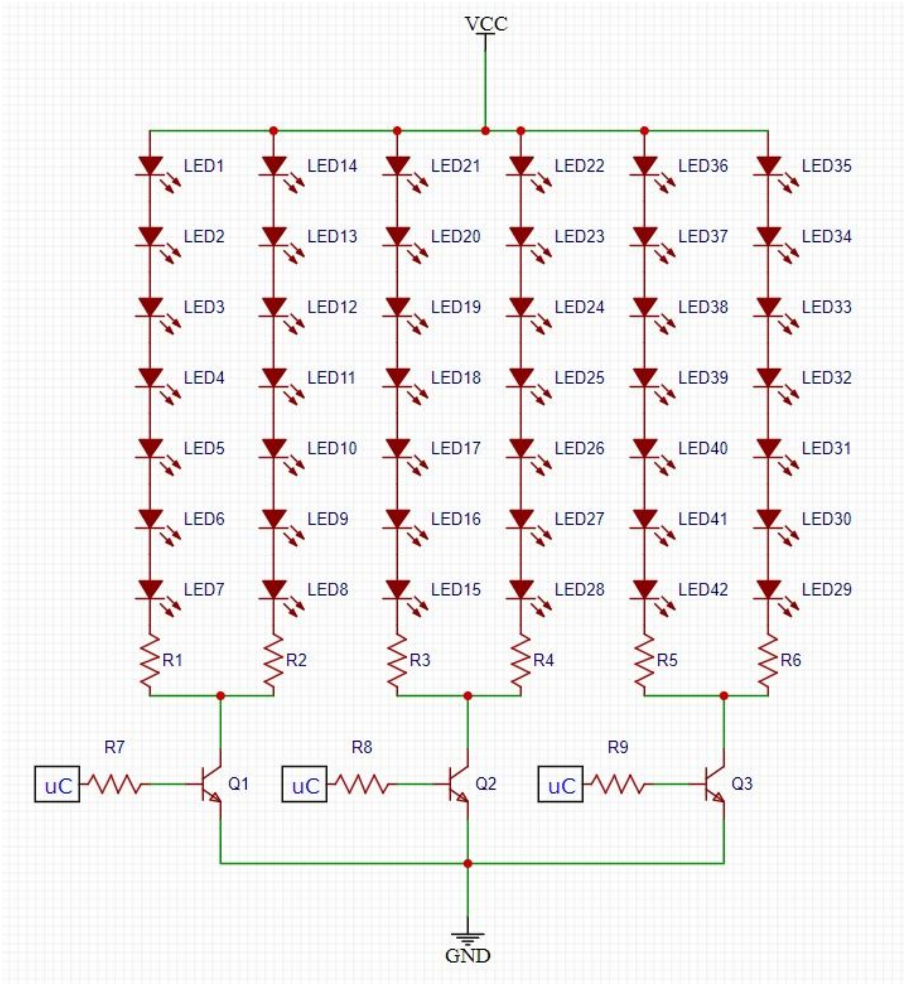

Fig S07. Electric circuit sized for the arrangement of RGB LEDs. In the figure, we can also see how each color of the RBG LED matrix is arranged for control by the microcontroller.

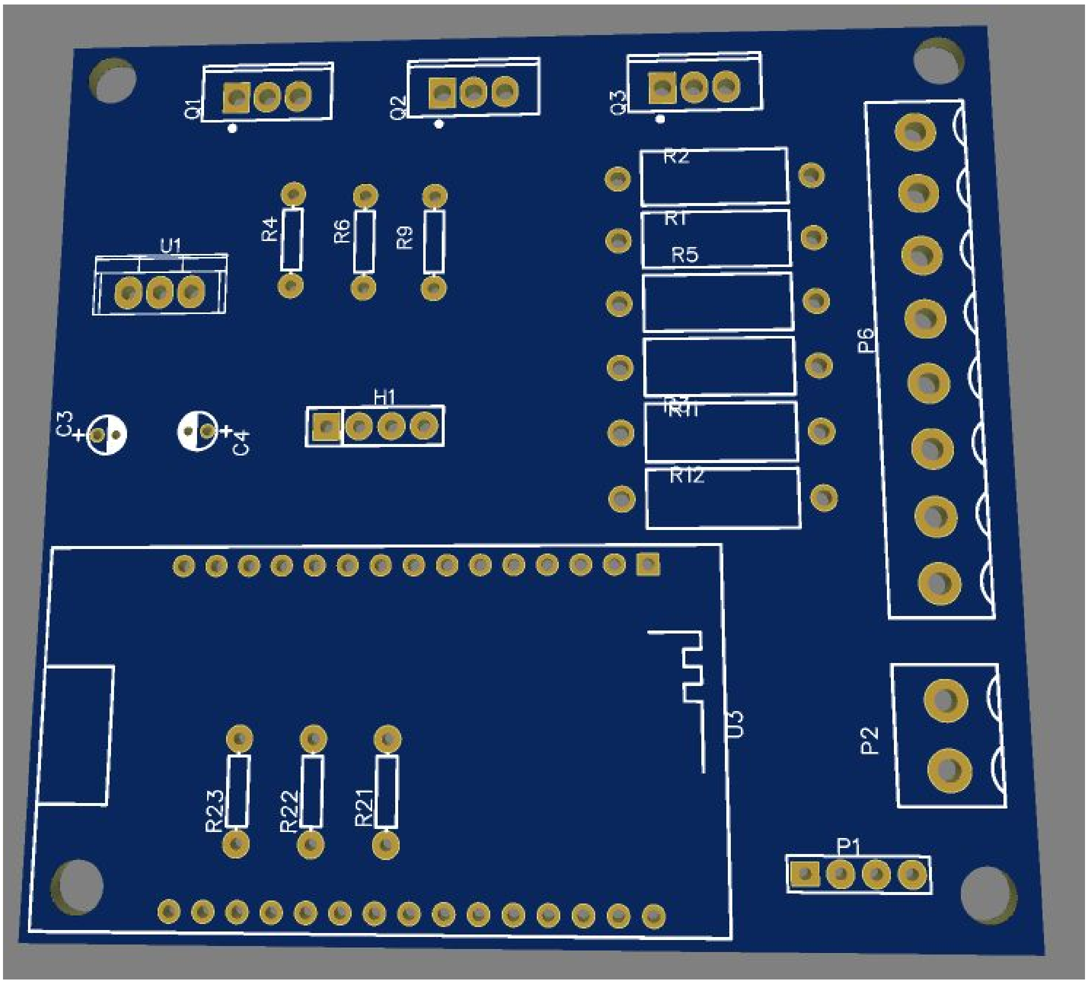

Fig S08. Electronic circuit board
