# Supplementary material for: A low-cost smart system for electrophoresis-based nucleic acids detection at the visible spectrum
Source: PLoS One. 2020 Oct 15;15(10):e0240536. doi: 10.1371/journal.pone.0240536 (PMC7561130; doi:10.1371/journal.pone.0240536)
Supplement: S1 Raw images — (PDF) [file pone.0240536.s002.pdf]

Original Images

The camera used to capture all the images was the Xiaomi MI 9 SE smartphone, which has the Sony IMX586 Exmor RS sensor. For this experiment, we only used the 48 MP camera with 1/2' and F/1.75, respectively, the size of the sensor and aperture. However, a minimum of 12 MP settings is sufficient for capturing gel images with the proposed LED device (PD One).

We used the Gimp image software (www.gimp.org) to extract, mark, and annotate the original images, which were compiled and then exported as a tiff file with LZW compression. Afterward, we build a single PDF compiled from several annotated tiff image files.

The gels' images are in the exact order according to the article's Fig 6. Each image presents a description of the dye used and the respective equipment for the dye excitation (a standard UV transilluminator and the PD One).

.

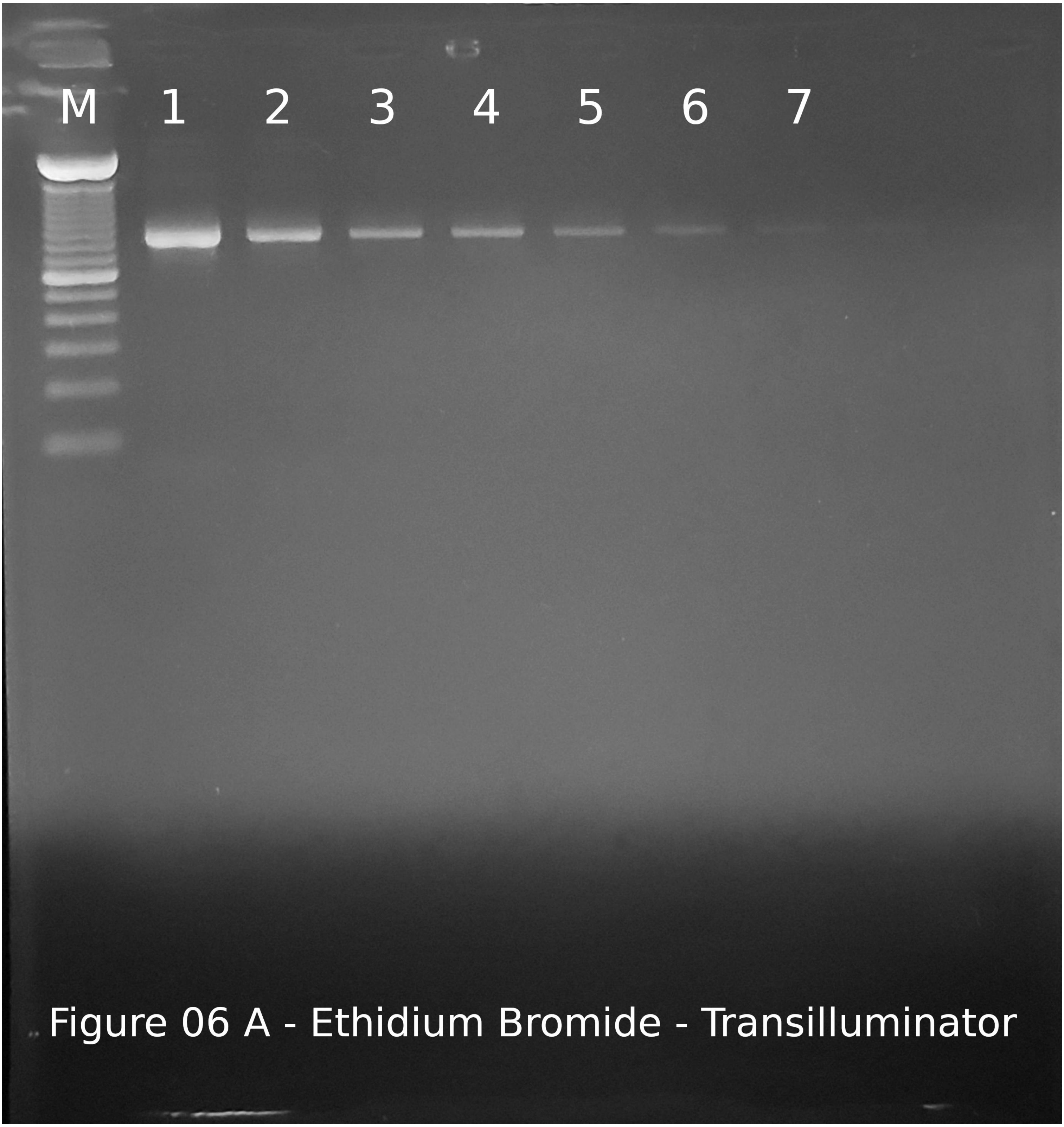

Figure 06 A - Ethidium Bromide - Transilluminator

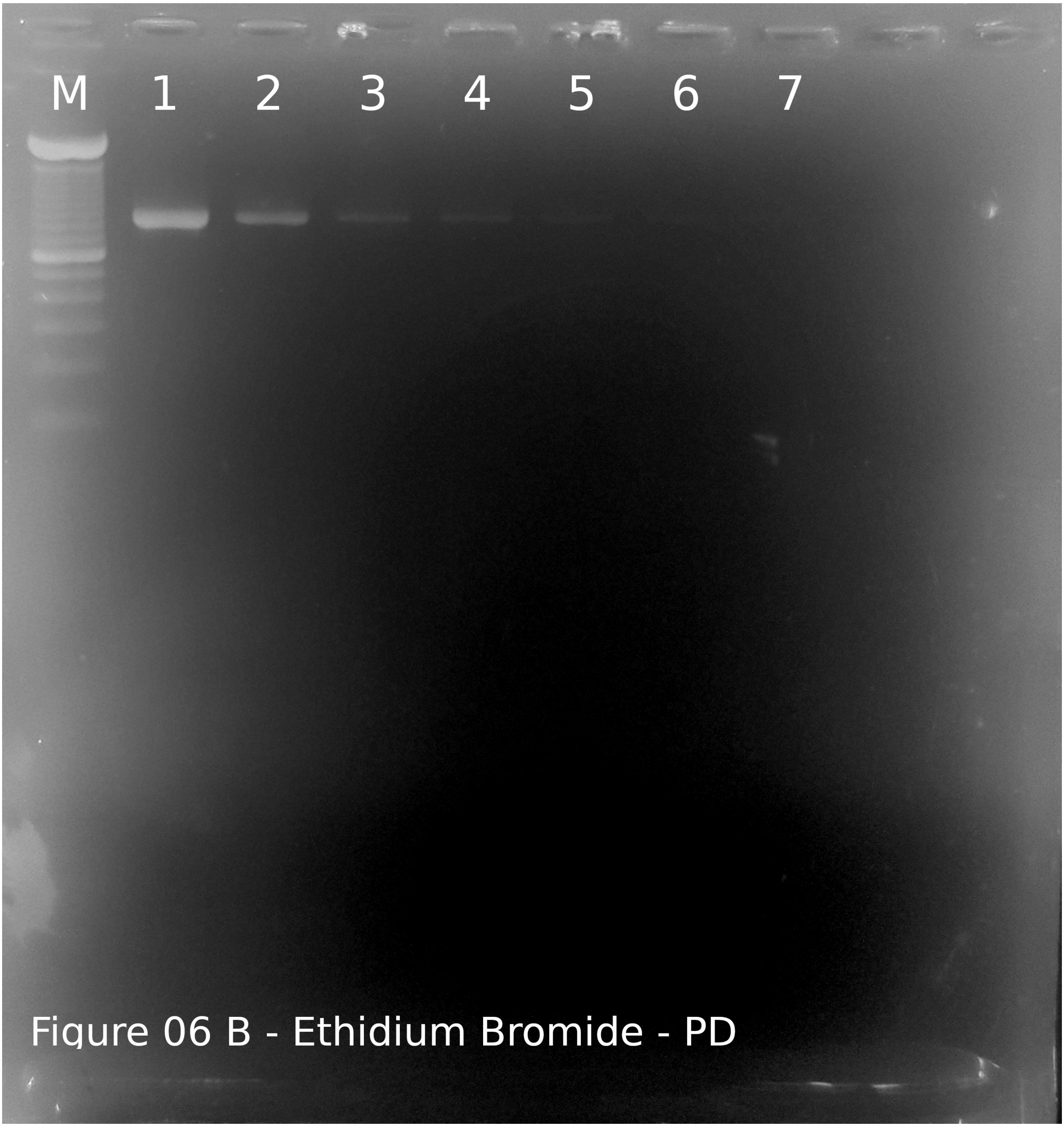

Figure 06 B - Ethidium Bromide - PD

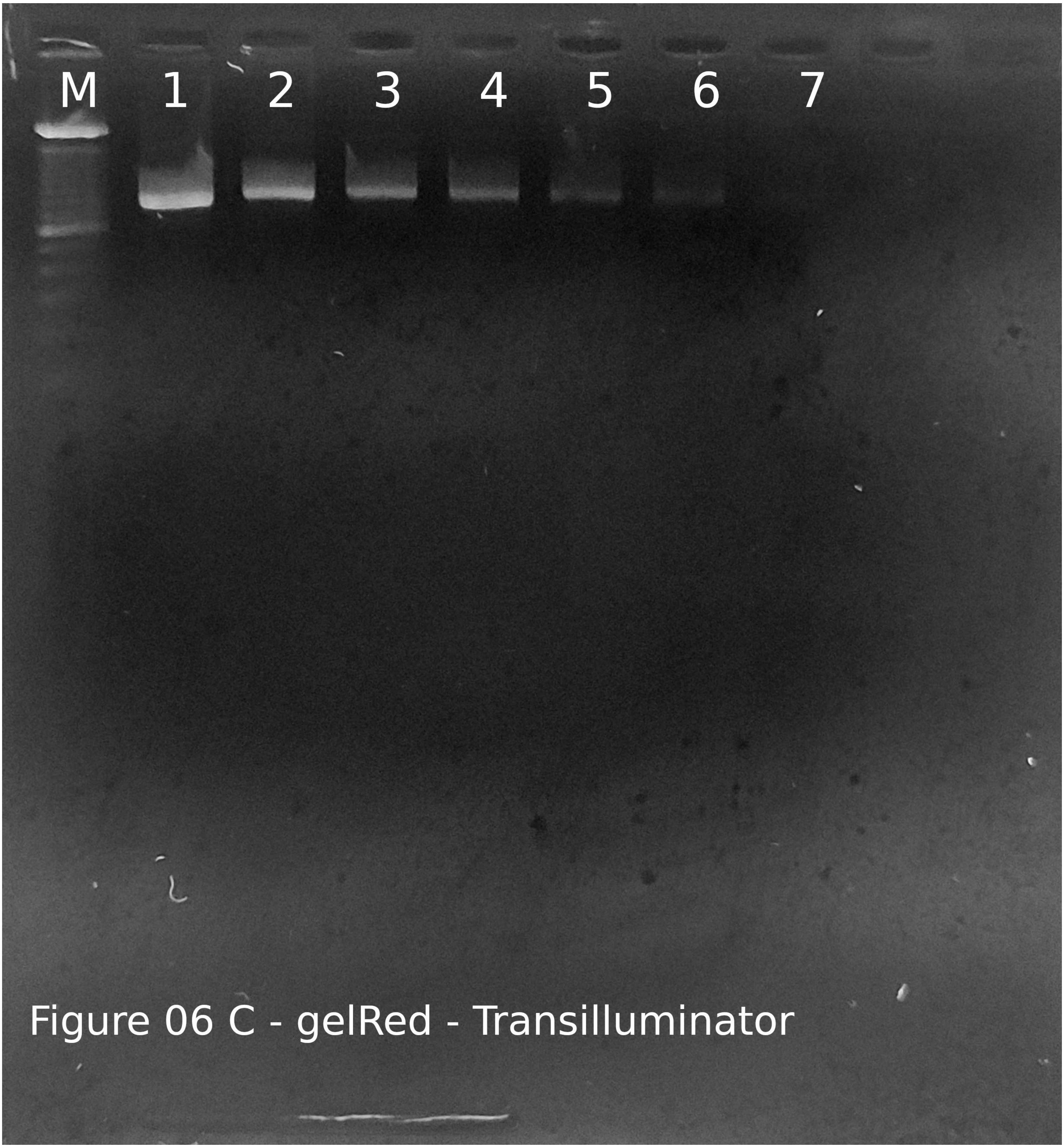

Figure 06 C - gelRed - Transilluminator

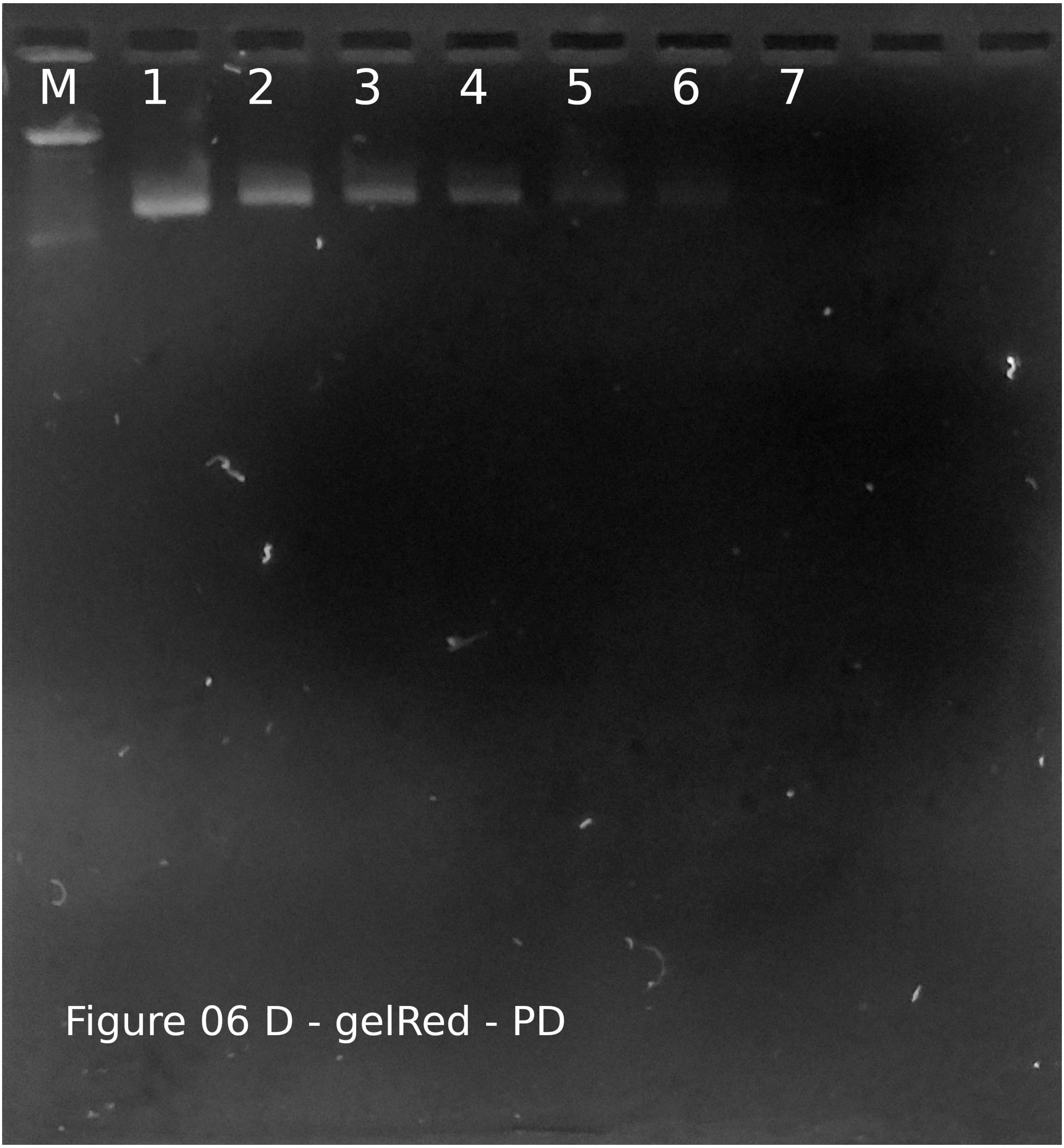

Figure 06 D - gelRed - PD

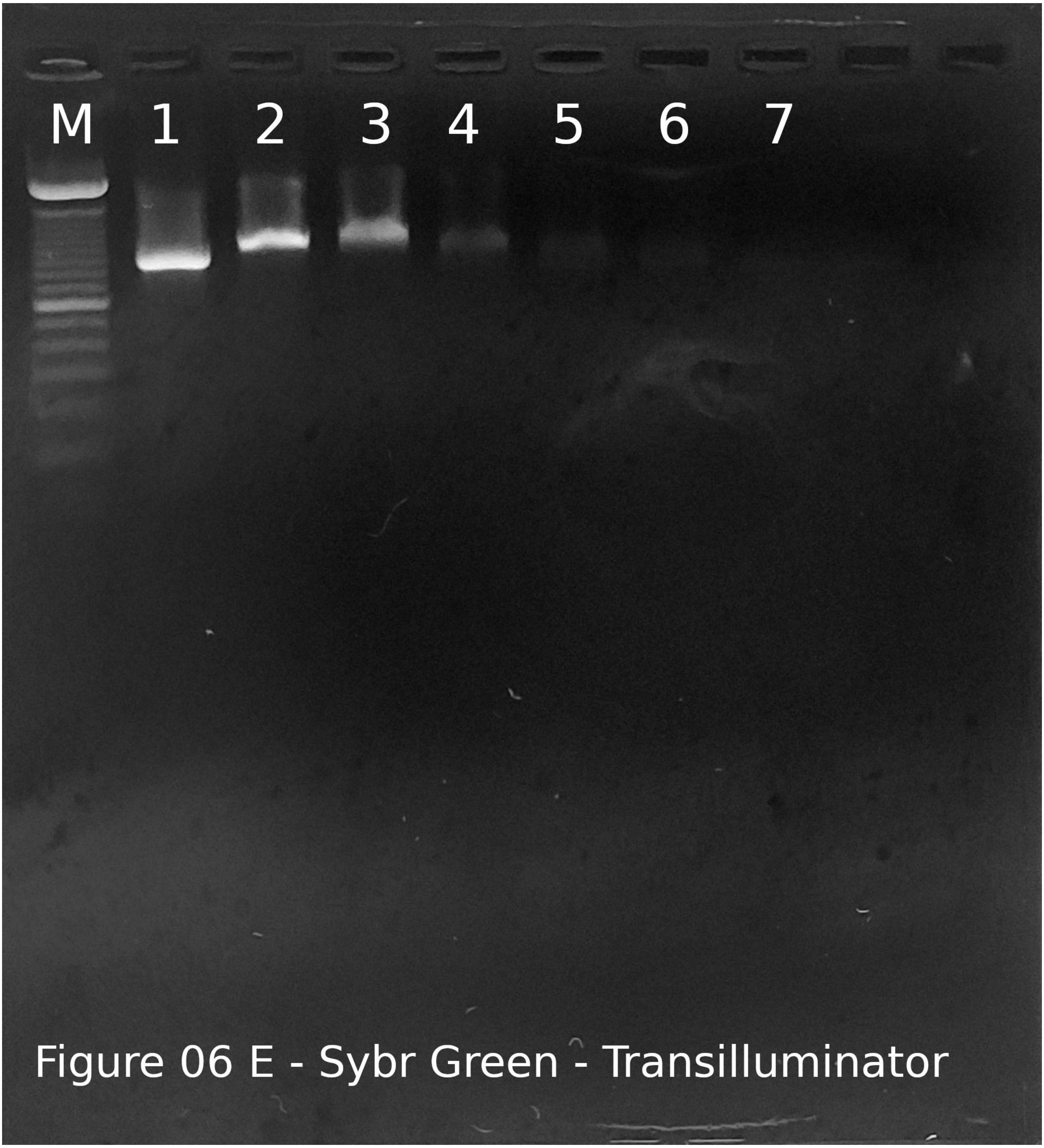

Figure 06 E - Sybr Green - Transilluminator

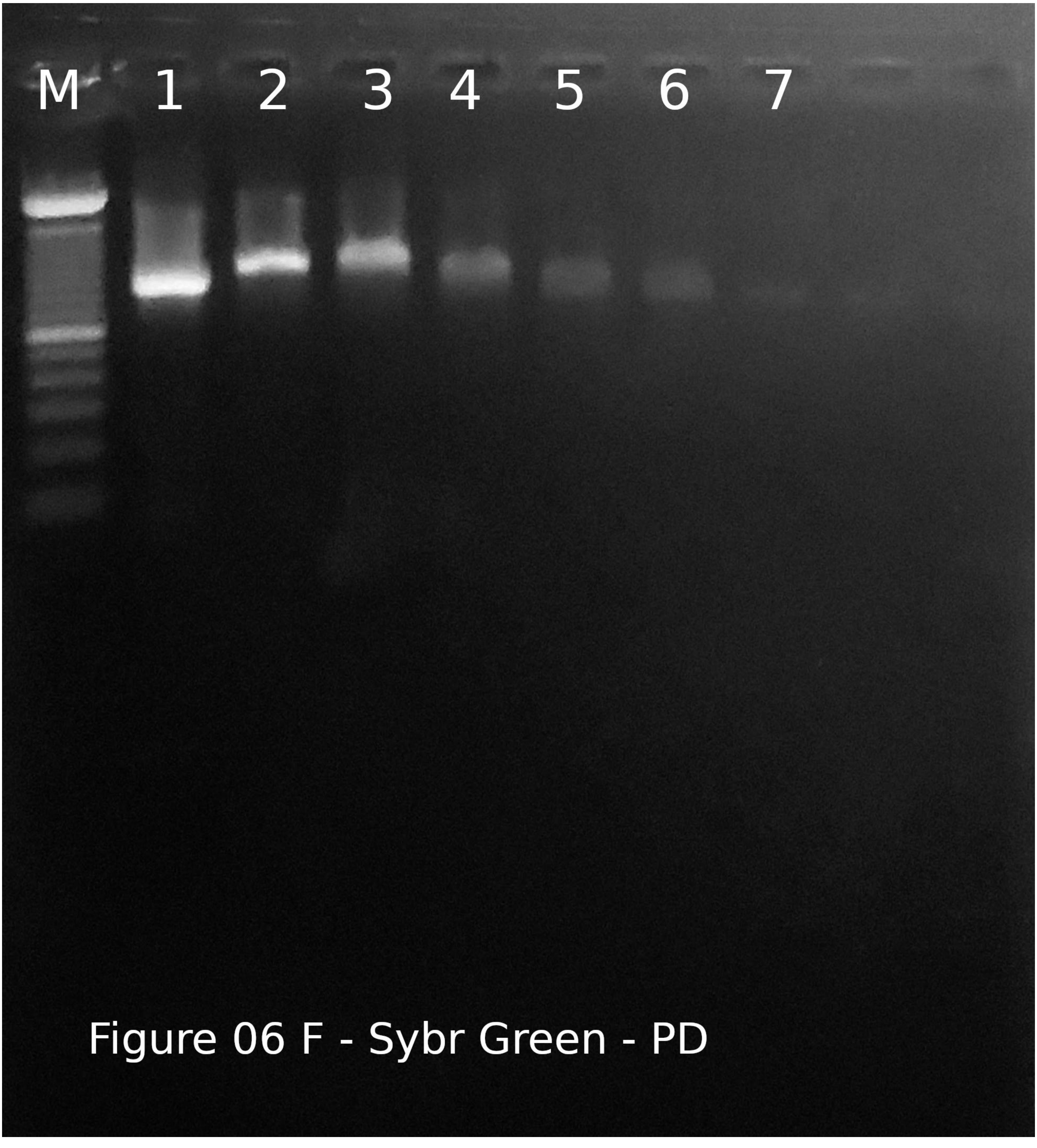

Figure 06 F - Sybr Green - PD
